# Outcomes of liver transplantation with thoracoabdominal normothermic regional perfusion: a matched-controlled initial experience in Spain

**DOI:** 10.3389/frtra.2023.1280454

**Published:** 2023-11-01

**Authors:** Luis Secanella, Felipe Alconchel, Javier López-Monclús, Enrique Toledo-Martínez, Oriana Barrios, Pablo Ramírez, Manuel Cecilio Jiménez-Garrido, Juan Carlos Rodríguez-Sanjuán, Mario Royo-Villanova, Gabriel Moreno-González, Laura Lladó

**Affiliations:** ^1^ Unidad HPB y Trasplante Hepático, Servicio de Cirugía General y Digestiva, Hospital Universitari de Bellvitge, Barcelona, Spain; ^2^ Servicio de Cirugía General y del Aparato Digestivo, Hospital Clínico Universitario Virgen de la Arrixaca (IMIB-Virgen de la Arrixaca), Murcia, Spain; ^3^ Servicio de Cirugía General y del Aparato Digestivo, Unidad de Trasplante Hepático, Hospital Universitario Puerta de Hierro, Majadahonda, Madrid, Spain; ^4^ Servicio de Cirugía General y del Aparato Digestivo, Hospital Universitario Marqués de Valdecilla, Santander, Spain; ^5^ Servicio de Medicina Intensiva, Coordinación de Trasplantes, Hospital Clínico Universitario Virgen de la Arrixaca (IMIB-Virgen de la Arrixaca), Murcia, Spain; ^6^ Servicio de Medicina Intensiva, Coordinación de Trasplantes, Hospital Universitari de Bellvitge, Barcelona, Spain

**Keywords:** asistolia, controlled donation after cardiac death, liver transplant, normothermic regional perfusion, heart transplant

## Abstract

Thoracoabdominal (TA) normothermic regional perfusion (NRP) should allow the safe recovery of heart and liver grafts simultaneously in the context of controlled donation after circulatory death (cDCD). We present the initial results of cDCD liver transplantation with simultaneous liver and heart procurement in Spain until October 2021. Outcomes were compared with a matched cohort of cDCD with abdominal NRP (A-NRP) from participating institutions. Primary endpoints comprised early allograft dysfunction (EAD) or primary non-function (PNF), and the development of ischemic-type biliary lesions (ITBL). Six transplants were performed using cDCD with TA-NRP during the study period. Donors were significantly younger in the TA-NRP group than in the A-NRP group (median 45.6 years and 62.9 years respectively, *p *= 0.011), with a median functional warm ischemia time of 12.5 min in the study group and 13 min in the control group. Patient characteristics, procurement times, and surgical baseline characteristics did not differ significantly between groups. No patient in the study group developed EAD or PNF, and over a median follow-up of 9.8 months, none developed ITBL or graft loss. Extending A-NRP to TA-NRP for cardiac procurement may be technically challenging, but it is both feasible and safe, showing comparable postoperative outcomes to A-NRP.

## 1. Introduction

Intending to solve the organ shortage in the donor pool, the number of controlled donations after circulatory death (cDCD) has increased over the last decade (1). After the withdrawal of mechanical ventilation (WMV) and a period of warm ischemia, livers are traditionally, recovered by rapid in-situ cold preservation, using the so-called super rapid recovery (SRR). However, this procurement method has been associated with a high incidence of early allograft dysfunction (EAD) and ischemic-type biliary lesions (ITBL) (2). Although limiting risk factors, such as donor age, warm ischemia, and cold ischemia, have improved these results (3), global outcomes remained worse than with donation after brain death (DBD), especially for primary non-function (PNF), re-transplantation, and biliary complication rates (4). Abdominal normothermic regional perfusion (A-NRP) has emerged as a useful tool to obtain liver grafts safely from cDCD donors by recovering liver function after a warm ischemia time (WIT) and allowing this function to be checked before transplantation (5). Heart transplantation teams have therefore proposed that we can expand the donor pool through cDCD. Contrary to the requirements of the lungs, the heart requires reoxygenation for its functional recovery and evaluation (6). Extending A-NRP to thoracoabdominal NRP (TA-NRP) can lead to short periods of uncontrolled warm ischemia, especially during the assessment of the heart, and can increase the risk of biliary damage. Thus, extension to TA-NRP concerns liver transplantation teams. Although several series have reported on heart transplants from cDCD, little has been reported on the outcomes of liver grafts recovered with hearts (7).

We aimed to compare the initial experience of liver outcomes in Spain following the simultaneous procurement of livers and hearts by cDCD using TA-NRP, compared with a matched cohort of livers recovered using A-NRP.

## 2. Methods

### 2.1. Study design

In this retrospective multicentric study, we evaluated the short- and long-term outcomes of livers recovered from all cDCDs by TA-NRP for simultaneous heart recovery. The study was conducted in Spain from January 2020 to October 2021 and included a minimum of 6 months follow-up. As a control group, we evaluated the outcomes of livers obtained from cDCD donors immediately before and after each case at the same institution. An institutional review board approved the study (PR425/21).

### 2.2. Donors

We considered all neurocritical patients as potential cDCD donors if they had a Glasgow Coma Scale score <5 points or their intensive care physicians considered the coma irreversible. After team evaluation and explanation to the family, we obtained informed consent for donation and premortem interventions according to the Spanish regulatory framework. This included the need for the following: heparinization (500–600 UI/kg); percutaneous (or surgical) cannulation of the femoral artery and vein; placement of an occlusive aortic balloon in the supraceliac aorta through the contralateral femoral artery to ensure isolation of abdominal perfusion during A-NRP; (8) and arterial line monitoring, usually in the left arterial artery, to ensure blockage of the supra-aortic branches during recovery (9). The functional WIT (WIT) starts when systolic blood pressure falls below 60 mmHg, followed by a mandatory 5-minute non-touch period once the blood pressure becomes undetectable. Death is then determined, and recovery procedures can start.

### 2.3. Normothermic regional perfusion

NRP takes 60–120 min to complete. After the declaration of death, the aortic balloon is filled, A-NRP starts, and cardiac surgeons perform a rapid sternotomy and dissection of the supra-aortic branches to clamp them (10). At that point, the aortic balloon is deflated and NRP is extended to the thoracic organs. To avoid cerebral oxygenation from collateral vessels of the thoracic aorta, the cephalad ends of each aortic arch vessel are cannulated and drained blood is returned to the venous reservoir for retransfusion (11). Once the heart spontaneously reverts to sinus rhythm, TA-NRP is weaned or decreased to <1 L/min according to the local protocol. Heart function is assessed by transesophageal echocardiography, Swan-Ganz catheter, and direct visual inspection. If the heart graft is accepted, the cardiac recipient surgery starts and once prepared, the aortic balloon is re-filled, or the thoracic aorta is clamped with the inferior cava to maintain A-NRP while the heart graft is retrieved under cold preservation. Blood samples are taken every 30 min from starting NRP to assess aspartate transaminase (AST), alanine transaminase (ALT), lactate, and hematocrit levels.

In this initial series for the validation of the technique, we accepted livers as transplantable grafts if they met the following criteria: warm ischemia time <30 min, transaminases peak <3 times normal value, cardiac weaning with minimal vasoactive drugs requirements and without venous overload, and good macroscopic aspect of the liver during the procedure, with excellent cold perfusion.

### 2.4. Outcome data

Data were obtained from the liver transplantation registry at each center. We collected clinical data about both the donor and recipient, including age, sex, transplant indication, Model for End-Stage Liver Disease (MELD) score, comorbidities, body mass index, pre-WMV intensive care unit (ICU) duration, vasoactive drugs use, laboratory liver function parameters, cause of death, and cannulation timing (premortem, pre-WMV, or postmortem, after the declaration of death). We also recorded details of the procurement and implantation procedures, such as total WIT (from WLST to the start of organ preservation), functional WIT (from donor systolic blood pressure <60 mmHg to the onset of organ preservation), NRP time, vasoactive drugs use during NRP, transaminase and lactate values during NRP, cold ischemia time (CIT), implantation time, piggy-back use, blood component transfusion, and anastomosis types (portal, arterial, and biliary). Finally, we reported postoperative outcomes, such as time for extubation, ICU stay, liver function (on days 1, 3, 5, and 7), arterial and biliary complications, reintervention, early graft loss or re-transplantation, mortality at 90 days, and long-term patient and graft survival.

Each patient who received a liver graft by TA-NRP cDCD was matched with two patients who received liver grafts by A-NRP cDCD at each center (the patient before and after each).

Our primary endpoint was the incidence of EAD and non-anastomotic cholangiopathy. We defined EAD according to the Olthoff criteria (12) and non-anastomotic cholangiopathy as the presence of non-anastomotic biliary strictures (in the absence of arterial thrombosis or stenosis) on magnetic resonance cholangiography performed 1 month after liver transplantation and repeated when clinically indicated during follow-up.

### 2.5. Statistical analysis

Categorical variables are presented as absolute numbers and percentages, whereas continuous variables are presented as medians and interquartile ranges (IQR). We compared the TA-NRP and A-NRP groups by χ^2^ or Fisher's F for categorical variables and by Mann–Whitney U or Student t-test for continuous ones. Patient and graft survival is assessed using the Kaplan–Meier method. Statistical analyses were performed using Stata® 13.1 (StataCorp®, 4905 Lakeway Drive, College Station, Texas 77845 USA).

## 3. Results

The four participating centers in Spain performed six liver transplantations using TA-NRP for grafts obtained from cDCD donors during the study period (Table 1). This included one patient each from Hospital Universitari de Bellvitge (Barcelona), Hospital Universitario Marques de Valdecilla (Santander), and Hospital Universitario Puerta de Hierro (Madrid), and three patients from Hospital Universitario Virgen de la Arrixaca (Murcia). Most donors were young (age <50 years, *n* = 5; 83%) and were not overweight or obese (body mass index <25 kg/m^2^, *n* = 5; 83%). All cases underwent premortem canulation and had short functional WITs (<20 min). We selected 12 liver transplantations with grafts obtained by A-NRP from cDCD as controls.

**Table 1:** Main features of liver transplantations from cDCD donors with TA-NRP for simultaneous heart donation.

	RECIPIENT				DONOR				VSL—NRP			
	Sex (Age)	Group	Indication	MELD (Clinical)	Sex (Age)	BMI (kg/m^2^)	ICU (days)	VAD	Cannulation	FWIT (min)	NRP (min)	VAD
Case 1	M(64)	0	HCC + HCV	8	M(48)	24.8	14	No	Premortem	10	138	No
Case 2	M(63)	A	OH (AOC)	16	F(29)	20.8	3	No	Premortem	13	23	No
Case 3	M(65)	0	OH + HCV	7	F(43)	20.8	1	No	Premortem	16	120	Yes
Case 4	M(60)	0	HCC + OH + HCV	12	M(30)	23.9	39	No	Premortem	12	146	No
Case 5	M(60)	0	HCC + HCV	28	M(54)	29.4	8	No	Premortem	10	110	No
Case 6	M(50)	0	OH	17	M(50)	25.7	2	No	Premortem	13	104	Yes

MELD, Model for End-Stage Liver Disease; BMI, body mass index; ICU, intensive care unit; VAD, vasoactive drugs; FWIT, functional warm ischemia time; NRP, normothermic regional perfusion; M, male; F, female; HCC, hepatocellular carcinoma; HCV, hepatitis-C virus; OH, alcohol.

### 3.1. Description of the series

**Figure 1:**
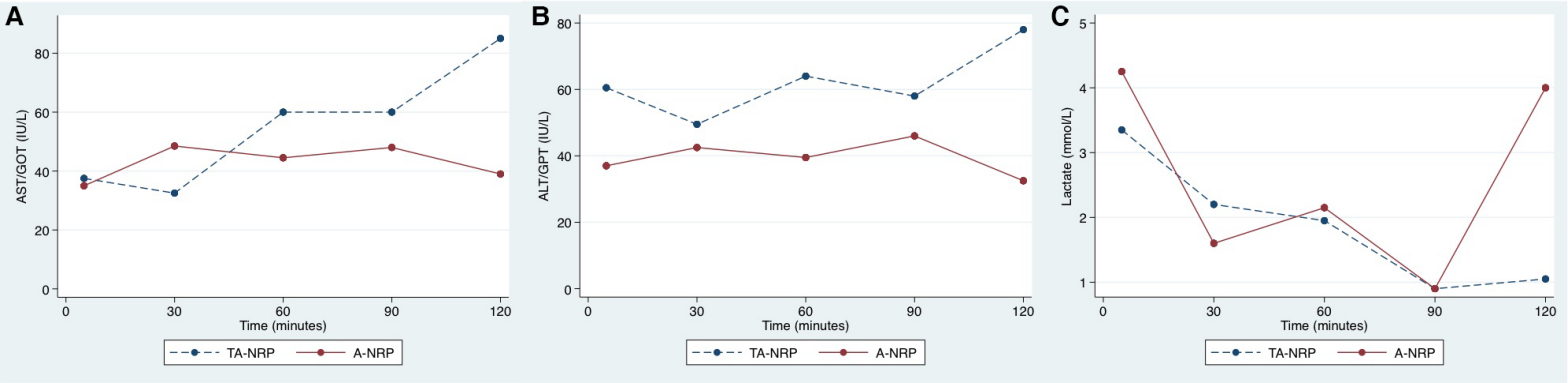
Evolution of AST (**A**), ALT (**B**) and lactate (**C**) during NRP. Note that transaminases levels tend to be higher in the TA-NRP group, specially before the first 30 min, once the weaning period has started.

**Figure 2:**
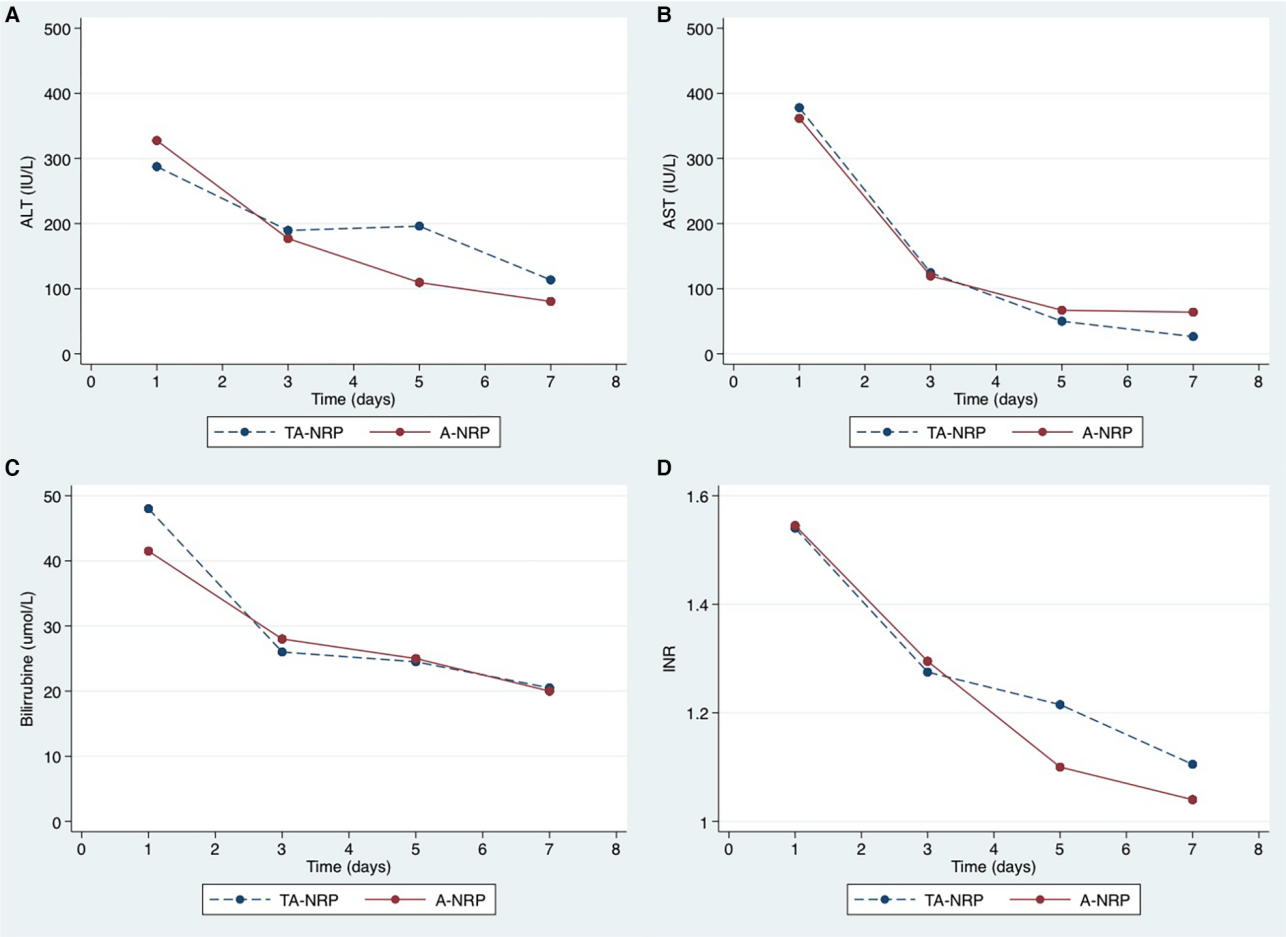
Postoperative evolution of transaminases (**A,B**), bilirrubine (**C**) and INR (**D**). The peak of transaminases occurred within the first 24 h post-transplantation, with a median of less than 400 IU/L. There were no differences in liver function recovery between TA-NRP and A-NRP groups.

Table 2 summarizes the descriptive analyses for the donors and recipients, together with technical issues. TA-NRP donors were significantly younger than A-NRP donors (median 45.6 vs. 62.9 years, respectively, *p *= 0.01) and had fewer comorbidities. No statistically significant differences existed between recipients, though we did observe a trend to allocate TA-NRP grafts to recipients with less comorbidity, and no differences existed in the WIT, CIT, or arterial ischemia time. Although we observed higher AST and ALT values in TA-NRP donors, these were not statistically significant (Figure 1 and Supplementary Table S1). The lactate curve was also comparable between groups, even during the weaning period. Postoperative liver function tests were optimal, with ALT and AST showing median peaks of <400 IU/L and rapid recovery of international normalized ratios (Figure 2 and Supplementary Table S2).

**Table 2:** Descriptive analysis of the A-NRP and TA-NRP groups.

	TOTAL	TA-NRP	A-NRP	*p*
	(*n *= 18)	(*n *= 6)	(*n *= 12)	
DONOR				
Age (years), median (IQR)	53.5 (43.1–68.7)	45.6 (31.0–50.1)	62.9 (52.2–69.2)	0.011
Sex (female/male), *n*(%)	5 (27.8%) / 13 (72.2%)	2 (33.3%) / 4 (66.7%)	3 (25%) / 9 (75%)	0.561
BMI (kg/m^2^), median (IQR)	25.8 (24.0–28.1)	24.3 (20.8–25.7)	27.7 (25.0–29.0)	0.111
ICU (days), median (IQR)	8.5 (4–12)	5.5 (2–14)	9.5 (4.5–11.5)	0.452
Cause of death, *n*(%)				0.236
CET	6 (33.3%)	4 (66.7%)	2 (16.7%)	
Stroke	6 (33.3%)	1 (16.7%)	5 (41.7%)	
Anoxia	6 (33.3%)	1 (16.7%)	5 (41.7%)	
Comorbidities, *n*(%)				
Diabetes	3 (16.7%)	0	3 (25%)	0.270
Cardiopathy	5 (27.8%)	1 (16.7%)	4 (33.3%)	0.439
CRA	5 (27.8%)	1 (16.7%)	4 (33.3%)	0.439
HipoTA, *n*(%)	1 (5.6%)	0	1 (8.3%)	0.667
VAD, *n*(%)	1 (5.6%)	0	1 (8.3%)	0.667
Median (IQR)				
Creatinine (umol/L)	66.5 (43–78)	50.5 (26–70)	67.5 (54–71)	0.373
ALT/GPT (IU/L)	36 (26–51)	33.5 (26–54)	36.5 (25–50.5)	0.851
AST/GOT (IU/L)	40 (29–42)	40 (33–55)	40 (29–41.5)	0.777
Bilirrubin (umol/L)	14.5 (10–16)	14.5 (12–18)	13 (8.5–16)	0.424
GGT (IU/L)	53.5 (45–74)	52.5 (45–61)	53.5 (43–79.5)	0.963
Alcaline Phosphatase (IU/L)	65.5 (57–80)	68 (58–83)	65.5 (50.5–77.5)	0.512
RECIPIENT				
Age (years), *median (IQR)*	60.9 (55.1–64.1)	62.0 (60.0–64.1)	60.8 (52.9–64.6)	0.708
Sex (female/male), *n(%)*	1 (5.6%) / 17 (94.4%)	0 / 6 (100%)	1 (8.3%) / 11 (91.7%)	0.667
BMI (kg/m^2^), *median (IQR)*	26.8 (24.2–28.9)	27.2 (24.2–29.0)	26.5 (24.4–28.8)	0.925
MELD (Clinical)*, median (IQR)*	16 (12–19)	14 (8–17)	16 (12–19)	0.452
Comorbidities, *n*(%)				
Abdominal surgery	4 (22.2%)	1 (16.7%)	3 (25%)	0.593
Diabetes	7 (38.9%)	1 (16.7%)	6 (50%)	0.199
HTA	4 (22.2%)	1 (16.7%)	3 (25%)	0.593
Hepato-renal syndrome	2 (11.1%)	1 (16.7%)	1 (8.3%)	0.569
Hospitalization (days)	1 (5.6%) / 1 (5.6%)	0 / 1 (16.7%)	1 (8.3%) / 0	0.569
NORMOTHERMIC REGIONAL PERFUSION				
Total WIT, median (IQR)	19 (16–24)	21.5 (16–26)	18.5 (16–21.5)	0.603
Functional WIT, median (IQR)	12.5 (10–15)	12.5 (10–13)	13 (10–15.5)	0.813
Time on NRP, median (IQR)	107 (90–122)	115 (104–138)	99 (90–120.5)	0.453
VAD on NRP, *n*(%)	2 (15.4%)	2 (33.3%)	0	0.144
IMPLANTATION				
Surgical time (min), median (IQR)	245 (229–309)	235 (222–250)	285 (229.5–309.5)	0.281
Piggy-back, *n*(%)	15 (83.3%)	5 (83.3%)	10 (83.3%)	0.730
Red blood cells, median (IQR)	4 (2–6)	4 (2–6)	4 (2–5)	0.737
Fresh frozen plasma, median (IQR)	0 (0–1)	0 (0–1)	0.5 (0–1.5)	0.469
Platelets, median (IQR)	0 (0–1)	0.5 (0–1)	0 (0–1)	0.715
Fibrinogen, median (IQR)	4 (1–6)	3.5 (0–6)	4 (2.5–5)	0.497
Reperfusion syndrome, *n*(%)	2 (11.1%)	1 (16.7%)	1 (16.7%)	0.569
CIT (min), median (IQR)	254 (185–300)	245 (185–300)	254 (185–295.5)	0.963
AIT (min), median (IQR)	45 (35–63)	60 (55–63)	41 (35–55)	0.357
AA (splenic/aorta), *n*(%)	3 (16.7%) / 1 (5.6%)	1 (16.7%) / 1 (16.7%)	2 (16.7%) / 0	0.676
PA with thrombectomy, *n*(%)	4 (22.2%)	1 (16.7%)	3 (25%)	0.593
BA (Kehr, bilioenteric), *n*(%)	1 (5.6%) / 1 (5.6%)	0	1 (8.3%) / 1 (8.3%)	1.000

A-, abdominal; AE, arterial anastomoses; AIT, arterial ischemia time; BA, biliary anastomoses; BMI, body mass index; CET, craneoencephalic trauma; CIT, cold ischemia time; CRA, cardio-respiratory arrest; ICU, intensive care unit; NRP, normothermic regional perfusion; PA, portal anastomoses; TA-, thoracoabdominal; VAD, vasoactive Drugs; WIT, warm ischemia time; INR, international normalized ratio.

### 3.2. Short-term outcomes

No patient developed EAD. One patient died on day nine from uncontrolled sepsis due to multi-resistant *klebsiella* species (the only early graft loss) and one patient required reoperation due to a biliary leak. However, five patients developed biliary complications (27.8%): four leaks (two in the TA-NRP group [33.3%] and two in the A-NRP group [16.7%]; *p *= 0.18), and 1 anastomotic stricture (5.6%) in the TA-NRP group (17.6%). The stricture was initially managed endoscopically, but it required a bilioenteric anastomosis in month eight.

### 3.3. Follow-up

Over a median follow-up of 9.8 months, the TA-NRP group experienced no mortality, graft losses, or non-anastomotic biliary strictures, but the A-NRP group had a graft lost due to ischemic cholangiopathy that needed re-transplantation in month seven (the same patient who needed reoperation for a biliary leak).

## 4. Discussion

To our knowledge, this is the first description and comparison of liver transplantation's short- and long-term outcomes using TA-NRP from cDCD with simultaneous heart recovery. This initial series of six transplantations in Spain revealed no EAD, mortality, graft loss, or ischemic cholangiopathy in the early post-transplantation period.

### 4.1. Normothermic regional perfusion in cDCD

The use of NRP in cDCD has reduced the high incidence of EAD/PNF and ischemic cholangiopathy classically associated with this type of donation. In a recent report of a Spanish national series comparing NRP with classic procurement by static cold storage (SCS) after asystole, the NRP group had significantly less EAD (81/545; 15%) compared with the SRR group (60/258; 23%), as well as fewer ischemic-type biliary lesions (6/545 [1%] vs. 24/258 [9%], respectively) (13). Furthermore, the incidence of hepatic artery thrombosis, re-transplantation, graft loss, and death, but not PNF, were significantly better in the NRP group. The Cambridge group also recently published a comparative study between SCS, NRP, and normothermic machine perfusion (NMP), reporting EAD in 21% of SCS procedures (19/97), which was non-significantly worse than for NRP (10/69; 14%) or NMP (7/67; 11%); non-anastomotic biliary strictures were also significantly more frequent in the SCS and NMP groups (22/97; 25%) than in the NRP group (12/67; 19%) (14). In the present series, we observed no allograft dysfunction, early re-transplantation, or ischemic cholangiopathy, having median ALT and AST peaks of 304.5 (max.1690) IU/L and 361.5 (max. 2,870) IU/L within the first week, respectively These results are consistent with the benchmarks for liver transplantation at 6 months (<20% biliary complications and <9% graft loss) (13). A meta-analysis published by De Beule et al. showed a lowered risk of EAD with NRP compared with in-situ cold preservation (RR 0.44; 95% CI 0.26–0.76) (15). Other strategies have been proposed to optimize livers from cDCD, such as ex-situ oxygenated perfusion (14), but those techniques are not established in Spain.

### 4.2. Thoraco-abdominal normothermic regional perfusion

When TA-NRP is proposed for liver and heart procurement, the two main concerns are prolonged WIT and the liver response to weaning NRP when checking heart function. In our series, the median functional WIT for TA-NRP was 12.5 min, comparable to that in the A-NRP group (13 min, *p *= 0.81), and lower than in other series with A-NRP. We consider that the use of premortem cannulation (cannulation before the withdrawal of ventilation) and a non-touch period of 5 min, coupled with the expertise of the cardiothoracic surgeons, produced this efficiency. The Spanish national experience in 2016, described by Hessheimer et al., reported a functional WIT of 19 min for postmortem cannulation and 12 min for premortem cannulation (16), with 3% of livers discarded due to altered laboratory values (21/775). In an international study by Muller et al., which compared outcomes between hypothermic static and oxygenated perfusion and NRP from cDCD, the functional WIT in the French group was 22 min with postmortem cannulation once femoral guidewires were pre-placed (17). Of the 226 donors in which NRP was initiated, 12 had to be discarded (12%) due to biochemical evolution during NRP, with ALT or AST levels increasing to over four times the normal limits. Experience with postmortem cannulation in the UK, described by Watson et al., reported no data about functional WIT, but did report the sole asystolic period to be 16 min and that 7% of donor's livers were discarded because of rising ALT or AST during NRP (18).

Although heart procurement by TA-NRP is well-established, especially in the UK, we know little of the outcomes of liver transplantation by TA-NRP from cDCD. The initial Spanish experience with heart procurement using TA-NRP has recently been published (10). This described four hearts transplanted successfully from young donors (age <45 years) with functional WITs between 8 and 16 min, fast restoration of spontaneous sinus rhythm (<1 min), and only two requiring low doses of vasoactive drugs. However, there has been only limited information about liver grafts in this context, with several procurement techniques described (19).

The UK experience included ten livers and nine hearts obtained by TA-NRP but does not report specific information about those livers (18). Croome and Daneshmand described their first experience with the combined procurement of three hearts and livers (20). They used SRR for the liver and isolated the thorax and abdomen by clamping the suprahepatic IVC and supraceliac aorta in the abdomen, then recovered the hearts and put them on the OCS Heart System in the context of a clinical trial. Although the procedure is described in detail, they provide no information about the outcomes of liver transplantation. Finally, Sellers et al. published the outcomes of 13 liver transplants using TA-NRP from cDCD (21), describing a median functional WIT of 21 min (range, 14–28) and a median NRP duration of 56 min (range, 42–71). Three patients developed EAD (23%), but none developed ischemic cholangiopathy (none underwent routine magnetic resonance cholangiopancreatography). In Spain, based on the experience with A-NRP for abdominal organ recovery, we chose to extend A-NRP to TA-NRP by clamping the supra-aortic vessels and deflating the aortic ballon (10). This requires that cardiothoracic surgeons perform a rapid sternotomy, for which we highly recommend premortem surgical or percutaneous cannulation through femoral vessels. After a few minutes recovery of heart beating, NRP must be weaned off to check the viability of the heart for possible episodes of hypotension or venous overload that could damage the liver graft (22).

One of the main challenges is the combination of TA-NRP with lung recovery. We know about the tolerance of the lungs to warm ischemia and their recovery through reoxygenation with mechanical ventilation, reflected by similar outcomes with DCD and DBD (23). Although pulmonary recovery with simultaneously A-NRP has been well described (24), the use of NRP for the lungs can lead to congestion of the pulmonary circulation and tissue edema. Therefore, once the TA-NRP extraction technique has been validated, the recovery of the lungs and heart has been proposed through a short period of cardiac preweaning and a shortened time in TA-NRP, limited to heart checking and the beginning of the procedure in the cardiac recipient. Lung transplantation outcomes using this procedure will have to be analyzed and published in the future (25).

The main ethical concern for the extension of PRN to the thorax was the possible reoxygenation of the brain. To avoid this, we must ensure before starting PRN that the supra-aortic branches are clamped. Once TA-NRP is established, the cephalic ends of each vessel of the aortic arch are cannulated and the drained blood is returned to the venous reservoir for retransfusion or vented to the atmosphere (26). This allows the evacuation of possible blood entry routes to the brain mainly through vertebral arteries (11).

### 4.3. Limitations

Despite providing important data for a knowledge gap in the literature, our study has several limitations. Of note, we included only a limited number of patients with a short follow-up period; consequently, this research only demonstrates the feasibility and safety of the procedure with optimal donor selection and highly coordinated transplantation teams. The excellent outcomes could be explained as follows: (1) the strict donor selection criteria required patients with mostly young ages, low comorbidity, and short ICU stays; (2) the exclusion of “risky” recipients, such as retransplantations, hepato-renal transplantation or portal vein thrombosis (27); and (3) the optimization of surgical strategies, such as lowering the CIT as much as possible (28). Further studies with more patients, longer follow-up periods, and heart donors with extended criteria will be needed to obtain strong evidence about graft quality after TA-NRP with suboptimal weaning times and that could affect the success of liver transplantation.

### 4.4. Conclusion

This initial study about the success of liver grafts by TA-NRP from cDCD shows that this is a feasible and safe procurement method in terms of early allograft dysfunction, patient and graft survival, and non-anastomotic biliary complications.

## Data Availability

The raw data supporting the conclusions of this article will be made available by the authors, without undue reservation.
