# Supplementary material for: Outcomes of liver transplantation with thoracoabdominal normothermic regional perfusion: a matched-controlled initial experience in Spain
Source: Front Transplant. 2023 Nov 1;2:1280454. doi: 10.3389/frtra.2023.1280454 (PMC11235216; doi:10.3389/frtra.2023.1280454)
Supplement: Supplementary file 1 [file Datasheet1.zip › Data Sheet 1_v1/72602 Suppl_Table_1.docx]

|  | Pre-ECMO  (n=18) | 5’  (n=18) | 30’  (n=18) | 60’  (n=18) | 90’  (n=10) | 120’  (n=4) |
| --- | --- | --- | --- | --- | --- | --- |
| ALT TA-NRP (IU/L) | 33.5 (26-54) | 60.5 (50-72) | 49.5 (37-64) | 64 (46-64) | 58 (43-102) | 78 (42-114) |
| ALT A-NRP (IU/L) | 36.5 (25-50.5) | 37 (22-49) | 42.5 (24.5-55) | 39.5 (24.5-53.5) | 46 (40-52) | 32.5 (25-40) |
| *p* | 0.851 | 0.025 | 0.426 | 0.058 | 0.173 | 0.121 |
| AST TA-NRP (IU/L) | 40 (33-55) | 37.5 (30-59) | 32.5 (30-58) | 60 (36-73) | 60 (43-113) | 85 (43-127) |
| AST A-NRP (IU/L) | 40 (29-41.5) | 35 (23.5-42) | 48.5 (34-59) | 44.5 (39.5-56.5) | 48 (38-50) | 39 (35-43) |
| *p* | 0.777 | 0.325 | 0.425 | 0.399 | 0.347 | 0.221 |
| Lactate TA-NRP (mmol/L) |  | 3.4 (1.1-4.8) | 2.2 (1-4) | 2.0 (0.8-3.1) | 0.9 (0.5-2.4) | 1.1 (0.2-1.9) |
| Lactate A-NRP (mmol/L) |  | 4.3 (1.6-7.8) | 1.6 (1.2-4.0) | 2.2 (0.9-4.2) | 0.9 (0.9-2.5) | 4 (3-5) |
| *p* |  | 0.303 | 0.743 | 0.453 | 0.459 | 0.121 |
| Table 3. Evolution of transaminases and lactate during NRP. Results are shown as medians and interquartile ranges. Abbreviations: ALT, alanine transaminase; AST, aspartate transaminase; ECMO, Extracorporeal membrane oxygenation; NRP, normothermic regional perfusion; TA-, thoracoabdominal. | | | | | | |
