# Supplementary material for: Outcomes of liver transplantation with thoracoabdominal normothermic regional perfusion: a matched-controlled initial experience in Spain
Source: Front Transplant. 2023 Nov 1;2:1280454. doi: 10.3389/frtra.2023.1280454 (PMC11235216; doi:10.3389/frtra.2023.1280454)
Supplement: Supplementary file 1 [file Datasheet1.zip › Data Sheet 1_v1/72602 Suppl_Table_2.docx]

|  | | | TOTAL  (n=18) | TA-PRN  (n=6) | A-PRN  (n=12) | *p* |
| --- | --- | --- | --- | --- | --- | --- |
| Extubation (hours), *median (IQR)* | | | 245 (120–660) | 155 (90–660) | 300 (150–630) | 0.574 |
| ICU stay (days), *median (IQR)* | | | 3 (2–4) | 3 (2–4) | 3 (2–3.5) | 0.806 |
| Liver function, *median (IQR)* | | |  |  |  |  |
|  | Day 1 | |  |  |  |  |
|  |  | Creatinine (μmol/L) | 85.5 (69–98) | 89.5 (83–118) | 75 (68–95) | 0.325 |
|  |  | ALT (IU/L) | 304.5 (209–625) | 287.5 (209–1072) | 327.5 (216–612) | 0.851 |
|  |  | AST (IU/L) | 361.5 (212–778) | 378 (273–1235) | 361.5 (205.5–625) | 0.851 |
|  |  | Bilirubin (μmol/L) | 46.5 (24–61 | 48 (24–60) | 41.5 (22.5–61) | 0.815 |
|  |  | INR | 1.54 (1.4–1.68) | 1.54 (1.29–1.6) | 1.55 (1.45–1.79) | 0.481 |
|  | Day 3 | |  |  |  |  |
|  |  | Creatinine (μmol/L) | 74.5 (64–109) | 68 (53–111) | 77.5 (70–97.5) | 0.640 |
|  |  | ALT (IU/L) | 177 (136–293) | 189.5 (143–372) | 177 (132–254) | 0.640 |
|  |  | AST (IU/L) | 120 (80–159) | 124.5 (80–138) | 119.5 (71–169) | 0.963 |
|  |  | Bilirubin (μmol/L) | 28 (18–54) | 26 (18–47) | 28 (14.5–54.5) | 0.779 |
|  |  | INR | 1.30 (1.16–1.36) | 1.28 (1.13–1.34) | 1.30 (1.17–1.41) | 0.574 |
|  | Day 5 | |  |  |  |  |
|  |  | Creatinine (μmol/L) | 70 (60–86) | 71 (60–102) | 70 (57.5–81) | 0.708 |
|  |  | ALT (IU/L) | 110.5 (95–179) | 196 (104–235) | 109.5 (81–144) | 0.134 |
|  |  | AST (IU/L) | 62.5 (40–68) | 50 (43–63) | 67 (36.5–83.5) | 0.190 |
|  |  | Bilirubin (μmol/L) | 25 (14–38) | 24.5 (39) | 25 (17–34.5) | 0.851 |
|  |  | INR | 1.17 (1.1–1.24) | 1.22 (1.16 -1.27) | 1.1 (1.06–1.22) | 0.091 |
|  | Day 7 | |  |  |  |  |
|  |  | Creatinine (μmol/L) | 63 (59–84) | 70 (62–84) | 61.5 (59–76) | 0.372 |
|  |  | ALT (IU/L) | 85.5 (50–109) | 113.5 (50–202) | 80.5 (54–91) | 0.261 |
|  |  | AST (IU/L) | 61.5 (26–75) | 26.5 (17–75) | 64 (31.5–72) | 0.512 |
|  |  | Bilirubin (μmol/L) | 20 (12–31) | 20.5 (12–31) | 20 (13.5–32.5) | 0.925 |
|  |  | INR | 1.09 (1–1.14) | 1.11 (1.09–1.23) | 1.04 (1–1.12) | 0.099 |
| Early allograft dysfunction, *n (%)* | | | 0 | 0 | 0 |  |
| Primary non-function, *n(%)* | | | 0 | 0 | 0 |  |
| Renal failure, *n (%)* | | | 4 (22.2%) | 2 (33.3%) | 2 (16.7%) | 0.407 |
| Arterial complications, *n (%)* | | | 0 | 0 | 0 |  |
| Biliary complications, *n (%)* | | | 5 (27.8%) | 3 (50%) | 2 (16.7%) | 0.176 |
|  | Leak | | 4 (22.2%) | 2 (33.3%) | 2 (16.7%) |  |
|  | Stricture | | 1 (5.6%) | 1 (17.6%) | 0 |  |
| Reintervention, *n (%)* | | | 1 (5.6%) | 0 | 1 (8.3%) | 1.000 |
| Hospital stay (days), *median (IQR)* | | | 16 (11–28) | 19.5 (13–28) | 15 (10.5–23) | 0.638 |
| Retransplantation, *n (%)* | | | 0 | 0 | 0 |  |
| Early graft loss, *n (%)* | | | 1 (5.6%) | 0 | 1 (5.6%) | 0.667 |
| 90-day mortality, *n (%)* | | | 1 (5.6%) | 0 | 1 (8.3%) | 0.667 |
| Table 4. Short-term outcomes of liver transplantations from cDCD with TA-NRP donors matched with A-NRP ones. Abbreviations: A-, abdominal; INR, international normalized ratio; NRP, normothermic regional perfusion; TA-, thoracoabdominal. | | | | | | |
